# ACEP: improving antimicrobial peptides recognition through automatic feature fusion and amino acid embedding

**DOI:** 10.1186/s12864-020-06978-0

**Published:** 2020-08-28

**Authors:** Haoyi Fu, Zicheng Cao, Mingyuan Li, Shunfang Wang

**Affiliations:** 1 School of Information Science and Engineering, Yunnan University, Kunming, 650500 China; 2 School of Public Health (Shenzhen), Sun Yat-sen University, Guangzhou, 510006 China

**Keywords:** Antimicrobial resistance, Antimicrobial peptide, Deep learning, Feature fusion, Visualization

## Abstract

**Background:**

Antimicrobial resistance is one of our most serious health threats. Antimicrobial peptides (AMPs), effecter molecules of innate immune system, can defend host organisms against microbes and most have shown a lowered likelihood for bacteria to form resistance compared to many conventional drugs. Thus, AMPs are gaining popularity as better substitute to antibiotics. To aid researchers in novel AMPs discovery, we design computational approaches to screen promising candidates.

**Results:**

In this work, we design a deep learning model that can learn amino acid embedding patterns, automatically extract sequence features, and fuse heterogeneous information. Results show that the proposed model outperforms state-of-the-art methods on recognition of AMPs. By visualizing data in some layers of the model, we overcome the black-box nature of deep learning, explain the working mechanism of the model, and find some import motifs in sequences.

**Conclusions:**

ACEP model can capture similarity between amino acids, calculate attention scores for different parts of a peptide sequence in order to spot important parts that significantly contribute to final predictions, and automatically fuse a variety of heterogeneous information or features. For high-throughput AMPs recognition, open source software and datasets are made freely available at https://github.com/Fuhaoyi/ACEP.

## Background

Antimicrobial resistance is one of our most serious health threats. Infections from resistant bacteria are now too common, and some pathogens have even become resistant to the multiple types of antibiotics [1]. Natural antimicrobials, known as host defense peptides or antimicrobial peptides (AMPs), defend host organisms against microbes, and most have shown a lowered likelihood for bacteria to form resistance compared to many conventional drugs [2]. AMPs have been demonstrated to kill Gram-negative and Gram-positive bacteria, enveloped viruses, fungi and even transformed or cancerous cells; thus, AMPs are considered as potential novel antimicrobial compounds [3]. Unlike the majority of conventional antibiotics, AMPs frequently destabilize biological membranes, form transmembrane channels and may also have the ability to enhance immunity by functioning as immunomodulators [4].

Over the last few decades, several AMPs have successfully been approved as drugs by FDA, which has prompted an interest in these AMPs. To aid researchers in novel AMP discovery, a variety of computational approaches are proposed for AMP recognition. Many incorporate machine learning algorithms or statistical analysis techniques, such as artificial neural networks (ANN) [5], discriminant analysis (DA) [6, 7], fuzzy k-nearest neighbors (KNN) [8], hidden Markov models (HM) [9], logistic regression (LR) [10, 11], random forests (RF) [6, 10], support vector machines (SVM) [6, 12] and deep neural network (DNN) [13].

To improve the recognition performance of AMPs, many popular feature extraction methods have been proposed. Basic amino acid counts over the N- and C-termini or the full peptide are used by the AntiBP2 methods [14]. The compositional, physicochemical and structural features are incorporated into the Pseudo-amino acid composition method [15, 16]. Constructing and selecting complex sequence-based features that capture information about distal patterns within a peptide are used in the evolutionary feature construction method [17, 18]. Physicochemical properties, such as charge, hydrophobicity, isoelectric point, aggregation propensity and more, are also used to encode sequences as numerical vectors [19].

In this paper, we improve existing AMP recognition technology. First, we introduce an amino acid embedding tensor that can map amino acids to tensors of real numbers automatically, which allows neural networks to discover similarity between amino acids. We use position-specific scoring matrices (PSSM) and these tensors to encode peptide sequences. The PSSMs contain the evolutionary information of sequences, which contributes to reducing the impact of amino acid variations in peptide sequences. Second, we design a new deep neural network, which has better performance on AMP recognition than existing methods. By applying the convolutional (Conv) layer and the ‘long short-term memory’ (LSTM) layer to our DNN, the model can effectively capture sequence features. Third, we discover some important motifs in sequences and build a ‘convolution and concatenation’ (CVCA) layer to fuse features by using the attention mechanism of natural language processing. Fourth, we ‘open’ the black box of ACEP model and explain the relationship between the patterns in deep neural network and the characteristics of sequences itself. Finally, we provide the source code and data on GitHub. The methods can be used to encode other types of protein sequences and improve the performance of sequence pattern recognition.

The superiority of DNN has been proven in many problems of bioinformatics, such as protein secondary structure prediction [20], protein folding recognition [21], membrane protein types prediction [22], drug discovery [23, 24], brain disease detection [25], etc. The tensor technique has been used for neural network data representation. A tensor is a container which can house data in *N* dimensions, along with its linear operations. In the paper, we refer to the data in neural network as tensors, which are generated during training neural network.

The attention mechanism has been used in natural language processing [26, 27], computer vision [28] and bioinformatics [29] to produce interpretable results for deep learning models. This strategy assigns different weights to each input feature, so that the model can focus on the most crucial features to perform better prediction.

## Results and discussion

### Model evaluation

We evaluate classification performance in terms of sensitivity (SENS), specificity (SPEC), accuracy (ACC) and Matthews Correlation Coefficient (MCC), which are defined using the number of true positive (TP), true negative (TN), false positive (FP) and false negative (FN) predictions.

1 $$ Sensitivity = \frac{{TP}}{{TP + FN}} \times 100\%  $$

2 $$ Specificity = \frac{{TN}}{{TN + FP}} \times 100\%  $$

3 $$ Accuracy = \frac{{TP + TN}}{{TP + FP + TN + FN}} \times 100\%  $$

4 $$ \begin{aligned} & MCC = \\ & \frac{{TP \times TN - FN \times FP}}{{\sqrt {(TP + FN)(TN + FP)(TP + FP)(TN + FN)}}} \end{aligned}  $$

We also make use of the receiver-operating characteristic (ROC) curve [30] to compare the performance of various methods. The ROC curve shows the performance of a classifier as the discrimination threshold is varied. In ROC curve figure, the x-axis represents the false positive rate and the y-axis represents the true positive rate. We calculate the area under the ROC curve (AUC) using the scikit-learn package in Python to evaluate performance in a quantitative, comparative setting. AUC ranges from 0.5 (corresponding to a random guess) to 1 (corresponding to the case when all predictions are correct).

### Model performance

Table 1 shows the classification performance, where columns 1 and 2 list training set and testing set, and columns 3-7 list SENS, SPEC, ACC, MCC and AUC. In particular, row 3 shows the performance of ACEP on the independent testing dataset, and the accuracy exceeds 93%, indicating that the model has a good generalization ability. The SENS, SPEC, ACC, and AUC values are all over 90%, and the MCC score is over 0.85. This row is used to compare with other AMP recognition methods. Row 5 shows recognition performance in a 10-fold cross-validation (CV) setting, where each of 10-folds is used once as a testing data with the model trained on the remaining 9-folds. The CV results represents the average performance of ACEP for out-of-sample data, and the relatively low standard deviation of ACC, MCC, and AUC indicates that our model has strong performance on approximately 90% of the data. Examining FN sequences from ACEP model on the testing set reveals 54 AMPs are missed (the detailed list is available in the Additional file Table).

**Table 1:** Model performance on different training and evaluation data partitions

Training set	Evaluation set	SENS(%)	SPEC(%)	ACC(%)	MCC	AUC (%)
Train	Tune	95.76	83.85	87.80	0.7582	96.67
Train	Test	93.39	90.44	91.92	0.8388	97.22
Train+Tune	Test	92.42	94.17	93.39	0.8663	97.63
All Data	All Data	98.26	99.66	98.96	0.9793	99.94
All Data	10-fold CV	91.37(± 1.05)	93.32(± 1.73)	92.46(± 0.87)	0.8474(± 0.01)	96.79(± 0.47)

Note: Performance is shown for ACEP model built and evaluated on the datasets listed in columns 1 and 2, respectively, on metrics listed in columns 3-7. The bottom line shows 10-fold CV performance, and the standard deviation is shown in parentheses

### Comparison with state-of-the-art methods

We compare ACEP model with 9 state-of-the-art machine learning methods proposed for AMP recognition between 2010 and 2018. In these methods, AntiBP2 used some new features based on terminal sequence composition; CAMP tried several common machine learning classifiers and a simple artificial neural network; iAMPpred introduced physicochemical characteristics and PseAAC; AMPScanner used deep learning technology. In Table 2, we list these methods chronologically, and line 10 shows our model. The bold in table 2 represent the best performance for a given metric.

**Table 2:** Performance comparison on the AMP dataset testing partition

Method	SENS(%)	SPEC(%)	ACC(%)	MCC	AUC(%)
AntiBP2	87.91	90.80	89.37	0.7876	89.36
CAMPr3-ANN	83.00	85.11	84.05	0.6813	84.05
CAMPr3-DA	87.07	80.75	83.91	0.6797	89.97
CAMPr3-RF	**92.69**	82.44	87.57	0.7553	93.63
CAMPr3-SVM	88.62	80.47	84.55	0.6933	90.62
iAMP-2L	83.99	85.86	84.90	0.6983	84.90
iAMPpred	89.33	87.22	88.27	0.7656	94.44
gkmSVM	88.34	90.59	89.46	0.7895	94.98
AMPScanner	89.88	92.69	91.29	0.8261	96.30
ACEP	92.41	**93.67**	**93.04**	**0.8610**	**97.78**

Note: Recognition performance on the testing dataset is shown for state-of-the-art methods (listed in column 1) on the metrics listed in columns 2-6. The best performance on a metric is marked in bold. Our deep neural network is shown in row 10

From Table 2, we can see that our method has the best performance in terms of SPEC, ACC, MCC and AUC. The random forests CAMPr3-RF achieves the highest SENS score (0.29% higher than our method). AMPScanner attains a similar performance (ACC and MCC values are reduced by approximately 2% and 0.04, respectively, compared with our method) due to using a Convolutional LSTM neural networks. The overall performance of the latest version of iAMPpred is also very good (ACC and MCC values are reduced by 4.7% and 0.1, respectively compared with our method). In addition, the AntiBP2 method limits the length of input sequences, so 211 test sequences are excluded when using this method for testing.

**Fig. 1:**
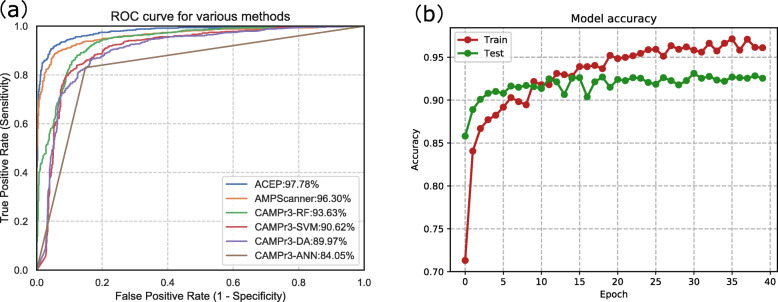
**a** ROC curves for the various methods compared, ordered by AUC. **b** Training history curves

Figure 1(a) compares the performance of various methods intuitively by plotting ROC curves. As shown in Table 2, AUC ranges from 84.05% to 97.78%. The AUC of ACEP (blue ROC curve) is approximately 1% higher than the suboptimal AMPScanner (orange ROC curve). The ROC curves of these methods are sorted in descending order according to AUC. CAMPr3-ANN does not provide the probability value of prediction results, so straight line is used to approximate the ROC curve.

To assess the stability of ACEP model in training process, we use training history data recorded by Keras to plot the curve of accuracy and training epochs, as shown in the Fig. 1(b). The red line is the accuracy of training data, and the green line is the accuracy of testing data. During training, the accuracy of training data and testing data increased steadily with the number of training epochs.

### Model visualization and analysis

To overcome the black-box nature of deep learning and enhance the interpretability of ACEP, here we visualize four important tensors in the neural networks, including the amino acid embedding tensor, the attention scores in the CVCA layer, the fusion tensor and the attention scores in the LSTM layer.

**Fig. 2:**
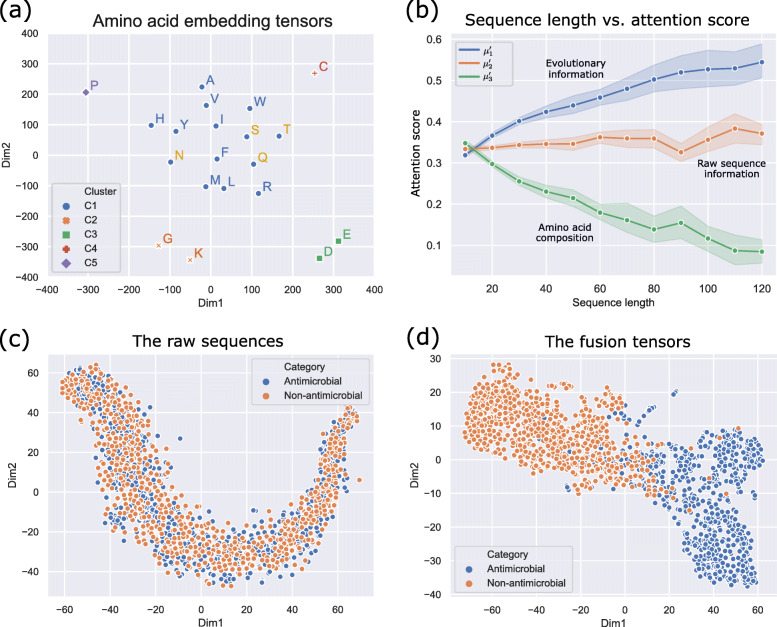
**a** The clustering of amino acid embedding tensors with evolutionary information. **b** The attention scores of evolutionary information, raw sequence information and supplementary information. **c** The raw sequences before being processed. **d** The fusion tensors after being processed

First, we extract the embedding tensor (*E*) carrying evolutionary information from the deep neural network. We use scikit-learn’s k-means algorithm [31] to cluster these amino acids into 5 clusters. Then, we use t-SNE algorithm [32] to reduce the dimensions of each amino acid tensor to 2D. Figure 2a shows the clustering results of 20 amino acids in *E* after dimension reduction. In this case, the distance indicates the similarity of amino acids. The amino acids with a shorter projection distance have more similar activation patterns, and these with longer projection distance have more differences.

In Fig. 2a, the negative amino acids aspartic acid (D) and glutamic acid (E) are clustered together because they both contain a negatively charged side chain. The amino acids with uncharged side chains, such as serine (S), threonine (T), asparagine (N) and glutamine (Q), are also close together in the cluster C1. Cysteine (C), which forms disulfide bonds, stands on its own in the top right, because it plays a unique role in structure formation or ligand interactions. It is perhaps unsurprising to see proline (P) is slightly isolated and distant. Proline-rich AMPs are shown to inactivate an intracellular biopolymer in bacteria without destroying or remaining attached to the bacterial cell membrane, and as such emerged as viable candidates for the treatment of mammalian infections [33]. These amino acid tensors are automatically generated by ACEP model during training.

ACEP model can fuse evolutionary information (EI), raw sequence information (RI) and supplementary information (SI) into a fusion tensor. The attention score (***μ***^***′***^) in CVCA layer can indicate which information ACEP model tends to pay attention to. We collect attention scores of 1424 sequences and plot the statistical graph of attention scores vs. sequence length, as Fig. 2b shows. We find that the attention score of EI increases with the sequence length because the phylogenetic information is more abundant for long sequences. And the attention score of SI decreases as the sequence length increases because amino acid composition only becomes available at short sequences; the attention score of RI remains almost unchanged because it’s not related to length. These attention scores are predicted by ACEP model, thus we speculate that the DNN model has learned a concept consistent with our cognition.

The fusion tensor (*F*_*meg*_) is a new representation of sequences generated by ACEP after integrating EI, RI and SI. In order to evaluate the quality of fusion tensors into which three types of information are fused, the 1424 sequences in testing dataset are projected onto a 2D space by using t-SNE. Figure 2c shows the raw sequences, and Fig. 2d shows the fusion tensors. The AMPs are represented by blue dots, and the non-AMPs are represented by orange dots. The fusion tensor forms two clusters in space, thus they are very effective to distinguish AMPs and non-AMPs.

**Fig. 3:**
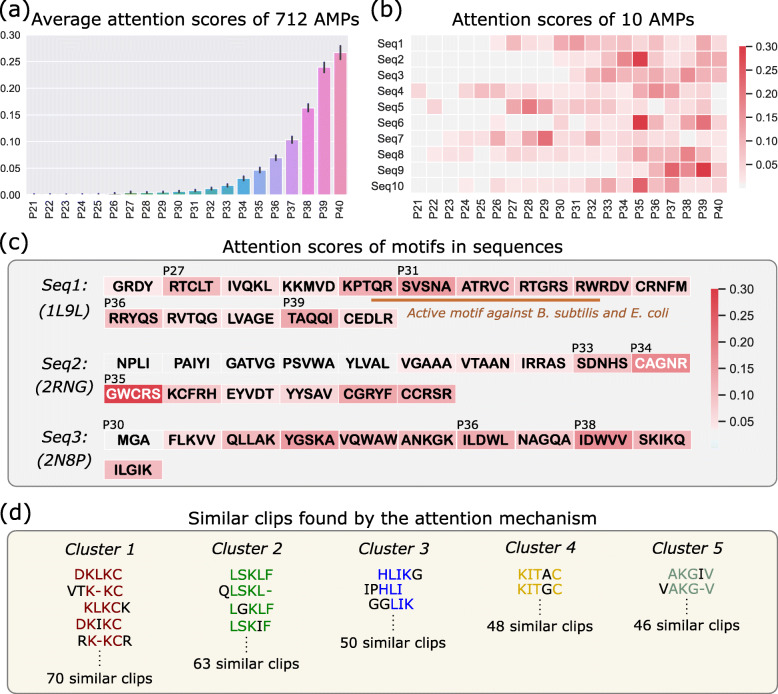
**a** The average attention scores calculated from 712 AMPs. **b** The heat map that visualizes the attention scores of different parts of 10 sequences. **c** The attention scores in sequences 1-3. **d** The similar clips found by the attention mechanism

The attention scores (***β***^***′***^) in the LSTM layer indicates which parts of a sequence are the most important (the length of sequences changes from 200 to 40 after passing through the pooling layer with a window of length 5). We calculate the average attention scores of 712 AMP sequences at 40 different positions and plot Fig. 3a. In Fig. 3a, the average attention score increases from 0 to 0.3 along sequence direction because the first half is invalid padding parts and the last half is the real sequence. The attention scores of the padding are close to 0s, indicating ACEP model can automatically ignore the padding and can effectively process variable-length sequences. We only list the results of P21 to P40, and the attention scores of P1 to P20 are all close to 0 (the complete results are shown in the Additional file Figure).

Next, we randomly select 10 AMP sequences and use ACEP model to calculate the attention scores of each part in the sequences. Through the attention mechanism, we can discover some important motifs in AMP sequences. Figure 3b shows the attention scores of 10 AMP sequences, and the brightness of blocks correspond to the attention scores. In subsequent calculations, the DNN will pay more attention to the parts with higher scores and ignore the parts with lower scores. Fig. 3c corresponds to the first three sequences in Fig. 3b, and the relatively high attention scores of some clips in the sequences imply that these parts may be active motifs and functional parts. For instance, the attention scores of sequence 1 have four peaks, which are the P27, P31, P36 and P39, and they exactly correspond to four *α*-*helix* structures in the sequence shown in PDB database (PDB ID: 1L9L) [34]. The subsequence ‘QRSVSNAATRVCRTGRSRW’ has some consecutive relatively high attention scores because it’s an active motif against *B. subtilis* and *E. coli* [35]. In sequence 2, the part P33-P35 with the highest attention scores corresponds to the structure of two *β*-*strands* connected by a *turn* (PDB ID: 2RNG) [36]. In sequence 3, the attention score of P30 is almost 0 due to no secondary structure, and the P36 and P38 are higher attention scores due to corresponding to two *α*-*helix* (PDB ID: 2N8P) [37]. In addition, we analyze the clips of AMP sequences with attention scores over 0.2 and find 67 clusters of similar clips. Taking cluster 1 for instance, the DNN model pays more attention to these clips that are quite similar, which implies that these clips may have some potential patterns contribute to peptide design. Based on the number of similar clips in each cluster, we ordered these clusters. In Fig. 3d, we show the first five clusters (the complete data is shown in the Additional Data).

### Comparison of modules

In ACEP model, the modules R1, R2 and R3 are used to process EI, RI and SI, respectively. We list all the combinations of these modules to compare the impact of each module on the overall performance of the system. In these combinations, when only one module is used, we disabled R4 (fusion module); when more than two modules are used, we integrate the output tensors of each module through R4. Table 3 shows the performance of the system in each case. In lines 1 to 3, the predicted performance in a single module is shown. Due to carrying EI, the R1 performs well in long sequences, with ACC exceeding 93%, but has poor performance for short sequences, with ACC about 89%. In lines 4 to 6, the performance integrated with two modules is shown. Because the amino acid composition contributes to the recognition of short sequences, the R1 + R3 is very effective for both long sequences and short sequences, with overall ACC exceeding 92%. The performance of the R2 + R3 is the worst for long sequences due to the lack of EI, with ACC about 88%.

**Table 3:** The performance of different modules

Module	Sequence length < 30			Sequence length ≥ 30			All sequences	
	ACC(%)	MCC		ACC(%)	MCC		ACC(%)	MCC
R1	89.74	0.7946		93.11	0.8623		91.29	0.8258
R2	91.03	0.8214		90.06	0.8013		90.58	0.8124
R3	89.09	0.7816		90.36	0.8077		89.67	0.7938
R1+R2	89.61	0.7926		92.50	0.8500		90.94	0.8188
R1+R3	91.03	0.8206		94.18	0.8846		92.48	0.8500
R2+R3	91.42	0.8284		88.37	0.7716		90.02	0.8018
R1+R2+R3	91.16	0.8236		94.34	0.8867		92.62	0.8527

## Conclusions

In this study, we developed a new protein classification algorithm for AMP recognition. In the encoding part, we use embedding tensor to capture hidden patterns between amino acids and integrate EI into sequence encoding. In the modeling part, the convolutional layer and LSTM layer are used to generate feature tensor; the attention mechanism is used to calculate the scores of each part in a sequence; the CVCA layer designed by us is used to fuse three types of feature tensors.

The latest comprehensive AMP data from APD database are used in training and testing of our DNN model. The results show that the performance on ACEP is better than the state-of-the-art methods. In addition, we overcome the black-box nature of deep learning and visualize some tensors of ACEP model, thereby discovering some similar amino acids and some meaningful motifs and explaining the working mechanism of the model. We offer all open source code of ACEP, including data preprocessing, model training and visualization. By loading pretrained weights, high-throughput AMP recognition can be easily performed on ordinary computers.

There are still several directions that can be further explored to advance this topic. ACEP model can accept and integrate a variety of heterogeneous information or features. At present, only EI and AAC are used in the research. In following research, some physicochemical features that are helpful to measure the antibacterial activity of sequences will be added to the model. The information of these biological processes will further expand the potential of ACEP model. It is also significant to integrate constantly updated AMP database. Relying on rich data, we can build a special predictor for AMPs with different activities and functions, and large-scale data are helpful to develop better algorithms.

In addition, ACEP model can encode sequences into a very effective multi-dimensional representation. If we use the DNN with SVM or Random Forest, it is likely to further improve recognition performance [38]. And it is also interesting to explore whether the model can identify the entire sequence of long AMP or the regions of sequences, and we will carry out this work in the future.

In conclusion, we hope that our method can help to find more AMPs and accelerate the research and development of AMP drugs. We also hope that ACEP model can be applied to a wider range of protein sequences analysis tasks.

## Methods

### Datasets

In this study, we hope that the training data can cover widespread AMPs and some newly discovered AMPs. In 2018, Daniel Veltri et al. [13] constructed a benchmark dataset of experimentally validated AMPs (released on AMPScanner website [39]). In the benchmark dataset, the positive samples consisted of 1778 AMPs that were active against Gram-positive and Gram-negative bacteria, which were screened from the largest comprehensive AMP repository APD [40]; the negative samples consisted of 1778 peptide sequences in cytoplasm, which were screened from UniProt [41] and filtered out antibiotic, antiviral, antifungal, effector or excreted characteristics. In addition, about 97.5% of the sequences were between 11AA and 100AA in length, and about 2.5% of the sequences were between 101AA and 200AA in length (some yet important antimicrobial proteins). The average length of all data is 34AA with a standard deviation of 22AA. The detailed sequence length distribution was shown in Figure in the Additional file. The dataset of 3556 peptide sequences was divided into three parts: 1424 for training, 708 for tuning and 1424 for testing.

### Encoding

#### Amino acid embedding

The 20 canonical amino acids can be classified according to their properties, and some important factors are charge, hydrophilicity or hydrophobicity, size, aggregation propensity and functional groups [42]. These properties can affect the function of amino acids, thus some amino acids with similar properties may also have similar functions. According to their side chains' *pK*_*a*_ values and charges carried at physiological pH (7.4), 20 standard amino acids can be divided into five groups, as shown in the Table 4.

**Table 4:** Groups of amino acids according to their properties

Groups	Amino Acids
Electrically Charged Side Chains(Positive)	R,H,K
Electrically Charged Side Chains(Negative)	D,E
Polar Uncharged Side Chains	S,T,N,Q
Hydrophobic Side Chains	A,I,L,M,F,W,Y,V
Special Cases	C,G,P

A single value to encode amino acids can’t reflect the similarity (distance) between amino acids [43]. To enable the DNN to automatically capture the hidden pattern of amino acids, we propose to use trainable tensors to represent individual amino acids. For each amino acid *u*_*k*_, we use a 64-dimensional embedding tensor to encode it, called the *E*_*k*_, and 20 amino acids are mapped to 20 embedding tensors as follows:

5 $$ {{u_{k}}} \to {{\boldsymbol{E}}_{k}}\;\;\;k =1,2,...,20  $$

**Fig. 4:**
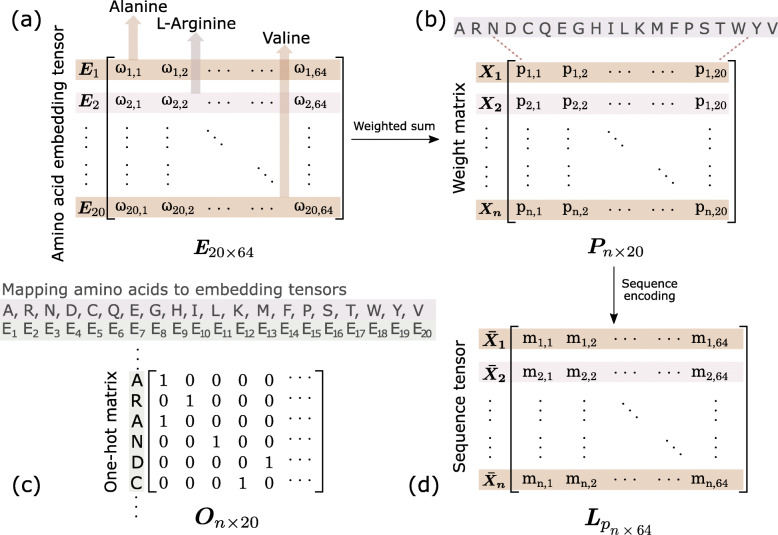
**a** The embedding tensor of 20 types of amino acids. **b** The *P* is the weight matrix of a sequence, where a row corresponds to a position in the sequence, columns represent 20 types of amino acids, and *n* denotes sequence length. **c** The *O* is a one-hot matrix, and each row in *O* is a one-hot vector converted from the corresponding numerical index of amino acids. **d** The *L*_*p*_ is the sequence tensor with evolutionary information

As Fig. 4a shows, we vertically stack the *E*_1_,*E*_2_,...,*E*_20_ into a trainable embedding tensor *E* and initialize it with a uniform distribution. During training, the *E* is updated constantly with the back-propagation algorithm. The advantage of using embedding tensor is that the similarity of amino acids can be measured by geometric distance between tensors.

#### Sequence tensor constructing

Due to the heredity and mutation of sequences in the process of evolution, the amino acid at each position of sequences may mutate into other amino acids. We obtain EI from the position-specific scoring matrix (PSSM) that contained the probability of occurrence of each type of amino acid at each position along with insertion or deletion. Hence, PSSM is considered as a measure of residue conservation in a given location [44].

We treat PSSM as the weight matrix of the sequence and rename it as *P*. The row corresponds to the position in the sequence and the column corresponds to 20 types of amino acids. The value *p*_*i*,*k*_ in *P* represents the weight of *k* amino acid in *i* position in the sequence. Thus, the EI for each amino acid is encapsulated in a vector of 20 dimensions, and the size of the *P* matrix of a peptide with *n* residues is *n*×20 as Fig. 4b shows.

For each sequence, the *P* matrix can be obtained during PSI-BLAST [45] search against Uniref50 database of protein sequences at online server POSSUM [46], and three iterations of searching at threshold e-value of 0.001 are set. Next we calculate the weighted sum of amino acids at each position in the sequence, called ${\bar X}_{i}$, the specific definition is as follows.

##### **Definition 1**

The *E* is the embedding tensor of 20 types of amino acid, and *E*_*k*_ is one of the *E*. The *p*_*i*,*k*_ is the weight of amino acid *k* at position *i*. The tensor ${\bar X}_{i}$ at each position of sequence *L* satisfies formula (6). A sequence with *n* residues can be encoded as ${{L}_{p}} = [{\bar X}_{1},{\bar X}_{2}, \ldots,{\bar X}_{n}]$ as follows:

6 $$ {{\bar X_{i}} = \sum\limits_{k = 1}^{20} {{p_{i,k}} \cdot {E_{k}}} \;\;\;k = 1,2, \ldots,20}  $$

where *i* represents the position index of the sequence, and subscript *k*=1,2,…,20 represents the numerical index of amino acids.

We call *L*_*p*_ the sequence tensor with EI. To facilitate the calculation of the DNN model, we define *L*_*p*_ as a *n*×64 tensor of ${\bar X}_{1},{\bar X}_{2}, \ldots,{\bar X}_{n}$ stacked vertically in order, as shown in Fig. 4d. In this case, the *L*_*p*_ can be quickly calculated using the formula (7), as shown below:

7 $$ {{\boldsymbol{L}}_{p}} = {{\boldsymbol{P}}_{n \times 20}} \cdot {{\boldsymbol{E}}_{20 \times 64}}  $$

Compared with the use of some fixed numbers encoding sequences, the use of the weighted sum of amino acid embedding tensors encoding sequences can solve the problem of residue variation in sequences. At the same time, the embedding tensor can find the similarity between amino acids, thus the DNN can have better generalization ability and help to develop more abundant patterns of sequences.

Next, we encode the raw sequences to the one-hot vectors as the second input of ACEP. The amino acids in each position of a sequence are represented by a 20-dimensional one-hot vector. For a sequence of length *n*, we construct an *n*×20 matrix *O*, as shown in Fig. 4c. Through replacing the *P* with the *O*, we can obtain *L*_*o*_, we call *L*_*o*_ the sequence tensor with RI, as shown in formula (8). The *O* matrix only carry the raw information of the sequence.

8 $$ {{\boldsymbol{L}}_{o}} = {{\boldsymbol{O}}_{n \times 20}} \cdot {{\boldsymbol{E}}_{20 \times 64}}  $$

In the above two methods of encoding sequences, the dimensions of tensors depend on the length of sequences. And the short sequences encoded as lower-dimensional tensors are easily ignored by DNN. Therefore, we add amino acid composition (AAC) as a supplementary information to improve the sensitivity of DNN to short sequences. AAC is a most frequently used feature descriptor that can capture global compositional information of peptides [47]. We compute the occurrences of 20 types of amino acids in the sequence, and the feature vector for the AAC descriptor is as follows:

9 $$ {{\boldsymbol{AAC}} = ({V_{1}},{V_{2}}, \cdots,{V_{a} }, \cdots {V_{20}})}  $$

where *V*_*a*_ denotes the occurrence number of the amino acid type *a*.

**Fig. 5:**
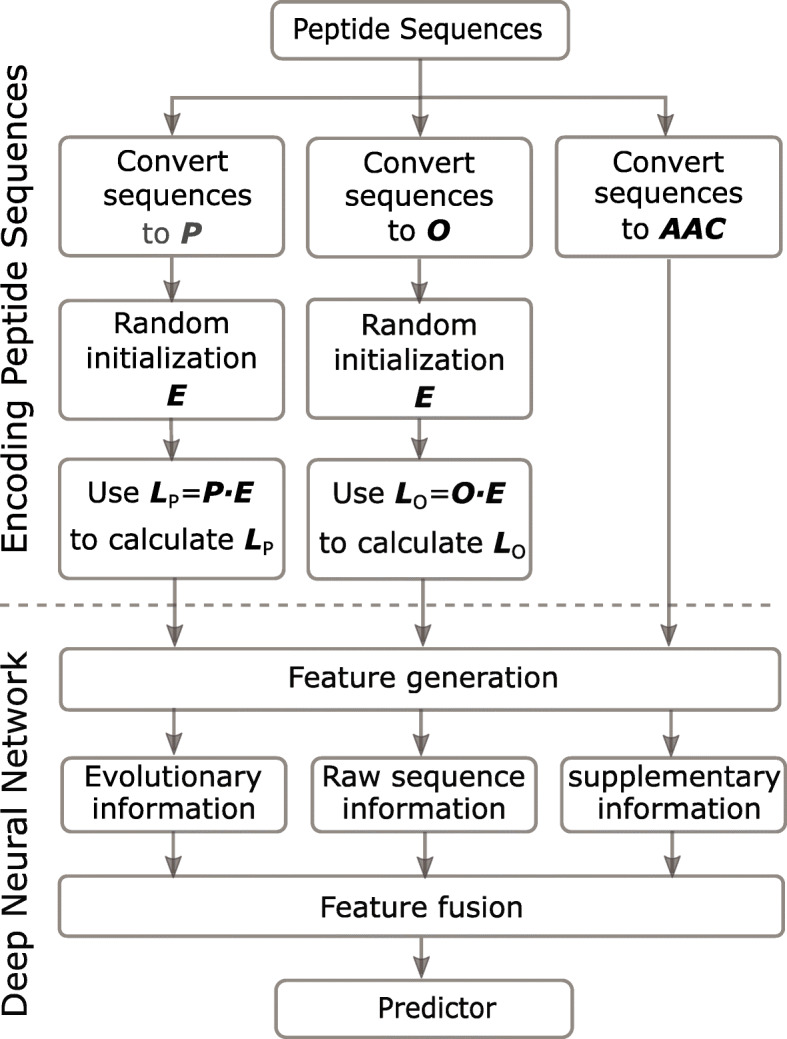
The flowchart of encoding sequences. The subsequent neural networks use the encoded tensors to generate the feature tensors with evolutionary information, raw sequence information and supplementary information

As shown in Fig. 5, each sequence is encoded as the *L*_*p*_, the *L*_*o*_ and the *AAC*. Then, they are sent to ACEP model to generate the feature tensors, and these feature tenors carry the EI, RI and SI. Finally, ACEP model integrates these feature tensors to predict results.

Although the PSSM profiles of short sequences contain almost no EI, we still convert short sequences into PSSMs in order that all length sequences can be represented by consistent descriptors, which makes the subsequent neural network easier to be trained. In addition, the neural network we designed can automatically select suitable descriptors, and these descriptors with insufficient information can be filtered by the attention mechanism in module R4.

### Architecture of proposed dNN

We design a new model based on deep learning called ACEP (Attention mechanism, Convolutional neural networks and Embedding tensor for antimicrobial Peptide recognition) to enhance the recognition of AMPs. We build ACEP model with the Keras framework [48] running on the TensorFlow [49] deep learning library (Detailed architecture of the model and the setting of parameters are shown in Figure in the Additional file).

**Fig. 6:**
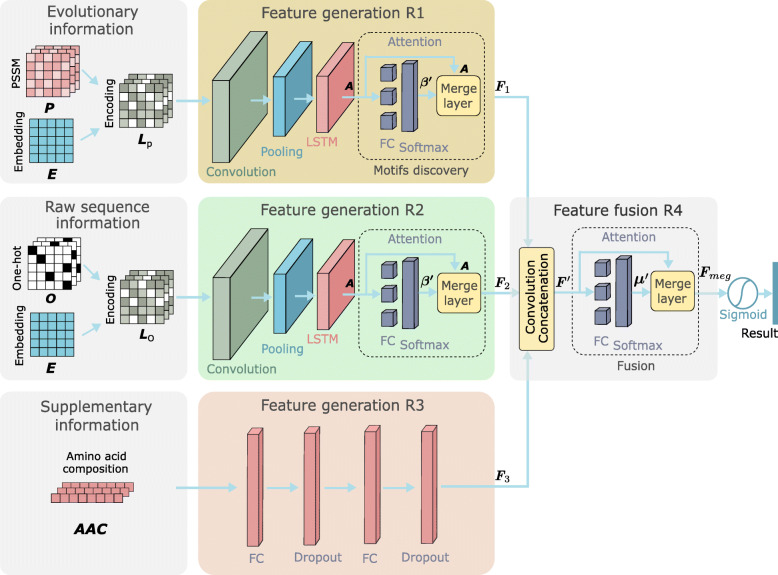
ACEP architecture consists of four module R1-R4. The R1-R3 are used to process different sequence information, and the R4 is used to fuse the feature tensors generated by the first three regions. In module R1, the Conv layer and the LSTM layer extract sequence features, and the attention layer predicts scores for different parts in sequences. In module R3, two fully connected layers are used to map 20-dimensional *AAC* vectors to 64-dimensional feature tensors. In module R4, we use the CVCA layer and the attention mechanism to fuse *F*_1_- *F*_3_ into *F*_*meg*_, then *F*_*meg*_ is passed to a Sigmoid function to predict results

ACEP model consists of four main functional modules. Module R1 and R2 are used to generate the feature tensor of *L*_*p*_ and *L*_*o*_, and module R3 is used to adjust the dimension of *AAC*, and module R4 is used to fuse the feature tensors generated by the first three modules, as Fig. 6 shows.

#### Feature extraction

In module R1, we use 1D convolution to automatically extract local features of *L*_*p*_, the Conv1D layer has 64 convolution kernels of size 16. And the max pooling layer downsamples sequences by sliding a non-overlapping window of length 5 and selecting the largest value. This layer prevents overfitting and speeds up calculations. Next, the LSTM layer with 64 units is applied to identify sequential patterns along the sequence direction. The LSTM is set to return a complete sequence, and the feature tensor at each time step is passed to next layer. The Dropout [50] in the LSTM layer helps prevent overfitting by randomly ignoring 30% of inputs. Each LSTM unit contains the input gate, output gate, hidden gate, forget gate, candidate cell gate and cell activation gate. These gates enable the model to remember or ignore the old information passed along the time step and prevent the gradient vanishing. In particular, the R1 and R2 are two independent modules with the same structure but have different parameters and inputs.

A growing number of biological studies point that different parts of an AMP sequence may be used for different purposes. Flexible termini may be important to disrupt membranes, and specific hydrophobic regions may serve as anchors to initiate interactions [51]. The attention scores (which can also be considered as weights) for different parts of a peptide sequence derived by the attention mechanism allow one to spot those important parts that significantly contribute to the final predictions [52]. Hence, the attention mechanism is a suitable technique to aid the discovery of the functional patterns of AMP sequences. In module R1, the attention layer and the merge layer work together to give different attention to different parts in the sequence. The attention layer predicts scores for each position in a sequence, and the merge layer merges the out of each position to form a new feature tensor using weighted sum. Finally, by training the model, the attention layer has ability to assign high scores to those parts that are more useful for recognition, and quickly ignores the padding characters.

**Fig. 7:**
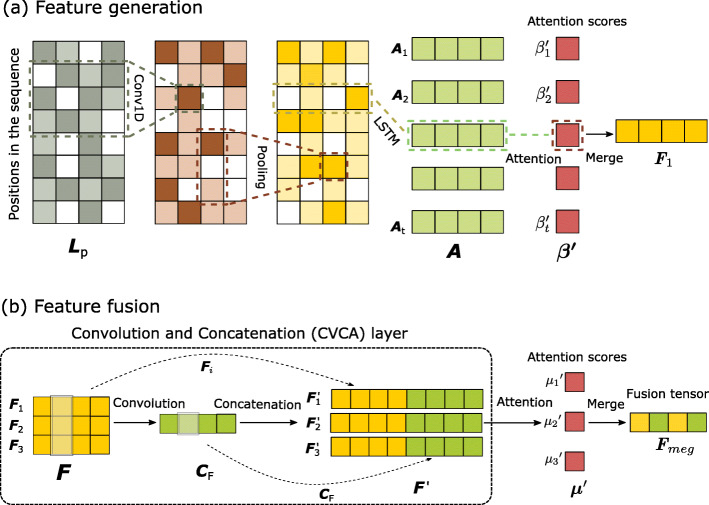
**a** The attention mechanism on the LSTM layer. **b** The convolution and concatenation layer

As Fig. 7a shows, we simplify the attention mechanism in natural language processing. The tensors returned in each time step in the LSTM layer are stacked vertically to form the tensor *A*. The attention layer calculates the score ***β*** for *A*, and these scores measure the importance of each position in a sequence, as follows:

10 $$ {\boldsymbol{\beta}} = {\boldsymbol{b}} + {{\boldsymbol{A}}}\boldsymbol{\omega}  $$

where ***ω*** is the weight of the fully connected layer in the attention module, *b* is the bias, and each fully connected layer shares the same parameters. Then, feed ***β*** into the Softmax layer and normalize it to ***β***^***′***^, as follows:

11 $$ \boldsymbol{\beta'} = {\mathop{Softmax}\nolimits}(\boldsymbol{\beta})  $$

The merge layer receives *A* and ***β***^***′***^ from the LSTM layer and the attention layer, then calculates the feature tensor *F*_1_ by weighted sum, as follows:

12 $$ {{\boldsymbol{F}}_{1}} = \boldsymbol{\beta'}{\boldsymbol{A}}  $$

In module R2, the feature tensor *F*_2_ is calculated in the same way as above. In addition, module 3 contains the fully connected layer with 64 units and the dropout layer, which is used to process *AAC*. The fully connected layer can map a 20-dimensional *AAC* vector to a 64-dimensional feature tensor *F*_3_ in order to fusing with other two feature tensors.

#### Feature fusion

In module R4, for the purpose of fusing the feature tensor *F*_1_- *F*_3_, we designed a ‘Convolution and Concatenation’ layer.

13 $$ \begin{array}{l} {\boldsymbol{F}} = {\mathop{Concat}\nolimits} ([{{\boldsymbol{F}}_{1}},{{\boldsymbol{F}}_{2}},{{\boldsymbol{F}}_{3}}])\\ {{\boldsymbol{C}}_{F}} = {\mathop{Conv}\nolimits} ({\boldsymbol{F}})\\ {{\boldsymbol{F}}_{i}}' = Concat([{{\boldsymbol{F}}_{i}},{{\boldsymbol{C}}_{F}}])\;\;\;\;\;i = 1,2,3\\ {\boldsymbol{\mu}} = {\boldsymbol{b}}+{\boldsymbol{F}}'\boldsymbol{\omega}\\ \boldsymbol{\mu}' = {\mathop{Softmax}\nolimits}(\boldsymbol{\mu})\\ {{\boldsymbol{F}}_{meg}} = \boldsymbol{\mu}' {\boldsymbol{F}} \end{array}  $$

As shown in the CVCA layer in Fig. 7b, *F*_1_- *F*_3_ constitute a tensor *F*, and a 1D convolution is used to convolve *F* to generate the convolutional tensor *C*_*F*_. Next, we respectively concatenate *C*_*F*_ with *F*_1_- *F*_3_ to form *F*1′- *F*3′, and vertically stack them into a tensor *F*^′^. Concatenating *C*_*F*_ with other three feature tensors makes attention scores more stable and effective. Then *F*^′^ is fed into the attention layer in order to generate the fusion score ***μ***, and ***μ*** is normalized to ***μ***^′^ by the Softmax function. Finally, we use attention scores to calculate the weighted sum of the three feature tensors (*F*_1_- *F*_3_). And *F*_*meg*_ stands for the fusion tensor containing the EI, RI and SI. In formula (13), ***ω*** is the weights of the fully connected layer, *b* is the bias, and each fully connected layer shares the same parameters.

#### Prediction

*F*_*meg*_ is fed into a Sigmoid function to predict classification results. We train ACEP model with 30 epochs and set the maximum length of the input sequences to 200AA, which can accept the longest sequence (183AA) in our dataset. For the sequences less than 200 in length, we fill *L*_*p*_ and *L*_*o*_ with 0s, making their dimensions 200×20. During training, the parameters in *E* are updated together with other parameters in ACEP model. The threshold value of the prediction probability > 0.5 is identified as AMP, and the probability ≤ 0.5 is identified as non-AMP.

### Amino acid clustering

**Fig. 8:**
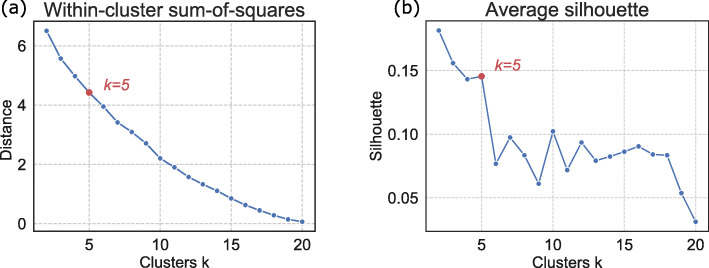
**a** The sum of squared distances under different *k* values. **b** The average silhouette under different *k* values

We extract the *E* from ACEP model, and cluster the embedding tensor of 20 types of amino acids using the k-means algorithm [31] in scikit-learn. To find the natural number of clusters, we calculate the average silhouette and the sum of squared distances under different *k* values, as shown in Fig. 8. The silhouette of a instance is a measure of how closely it is matched to data within its cluster and how loosely it is matched to data of the neighboring cluster, i.e., the cluster whose average distance from the datum is lowest [53]. The sum of squared distances measures the distance between the sample and the cluster center.

We draw the within-cluster sum-of-squares curve and the silhouette curve to determine the real *k* value. We expect the silhouette value of *k* to be as large as possible on the premise that the sum of squared distances is as small as possible. In Fig. 8, it can be noticed that the value of the silhouette is the largest when *k*=2, but the sum of squared distances is also very large, approximately 6.7, so the datum instances are far from the cluster center. As the trade-off between the silhouette and the sum of squared distances, we choose *k*=5 as the cluster numbers.

### Model tuning and cross-validation

By using the scikit-learn API provided by Keras, we package ACEP as a scikit-learn model to optimize hyperparameters. The RandomizedSearchCV of scikit-learn [54] is used to search optimal hyperparameters. The tuning step uses only the training dataset and the tuning dataset. After the hyperparameters are selected, the training model is established by combining the training dataset and the tuning dataset, and the performance is evaluated on the testing dataset.

We use CV to estimate how accurately our predictive model will perform in practice. Specifically, we split all the data (training, tuning and testing dataset) into *k* folds (*k*=10), a single fold is retained as the validation data for testing the model, and the remaining *k*−1 folds are used as training data. The CV process is then repeated *k* times, with each of the *k* folds used exactly once as the validation data. The *k* results can be averaged to produce a single estimation. During CV, the hyperparameters that were selected via model tuning are not changed. In summary, CV averages the measures of fitness in prediction to derive a more accurate estimate of model prediction performance.

## Supplementary information

**Additional file 1** **Figure S1.** Sequence length distributions of AMPs and non-AMPs. **Figure S2.** The shapes and connections of each layer in ACEP model. **Figure S3.** The attention scores of different parts of the sequences. **Table S1.** False negative AMP sequences.

## Data Availability

Open source software and datasets are made freely available at https://github.com/Fuhaoyi/ACEP. Using these codes, you can perform high-throughput AMP predictions, reproduce paper experiments and visualize experimental results.

## Abbreviations

AAC: Amino acid composition
ACC: Accuracy
AMP: Antimicrobial peptide
AUC: Area under the ROC curve
Conv: Convolution
CV: Cross validation
CVCA: Convolution and Concatenation
DNN: Deep neural network
EI: Evolutionary information
LSTM: Long short-term memory
MCC: Matthews Correlation Coefficient
PSSM: Position-specific scoring matrix
ROC: Receiver operating characteristic
RI: Raw sequence information
SENS: Sensitivity
SPEC: Specificity
SI: Supplementary information
t-SNE: t-distributed stochastic neighbor embedding
