# Supplementary material for: ACEP: improving antimicrobial peptides recognition through automatic feature fusion and amino acid embedding
Source: BMC Genomics. 2020 Aug 28;21:597. doi: 10.1186/s12864-020-06978-0 (PMC7455913; doi:10.1186/s12864-020-06978-0)
Supplement: Supplementary file 1 — Additional file 1 Figure S1. Sequence length distributions of AMPs and non-AMPs. Figure S2. The shapes and connections of each layer in ACEP model. Figure S3. The attention scores of different parts of the sequences. Table S1. False negative AMP sequences. [file 12864_2020_6978_MOESM1_ESM.pdf]

Additional file for

ACEP: improving antimicrobial peptides recognition through automatic feature fusion and amino acid embedding

Haoyi Fu [1], Zicheng Cao [2], Mingyuan Li [1] and Shunfang Wang [1,*]

[1]School of Information Science and Engineering, Yunnan University, Kunming 650500, China
[2]School of Public Health (Shenzhen), Sun Yat-sen University, Guangzhou 510006, China.
[*]To whom correspondence should be addressed.

1 Length distributions of sequences

Sequence length distributions are shown for the training set (top), tuning set (middle), and testing set (bottom) partitions in Figure S1. All the sequences come from a benchmark dataset constructed by Veltri *et al.* (2018) using data from the APD (Wang *et al.*, 2015).

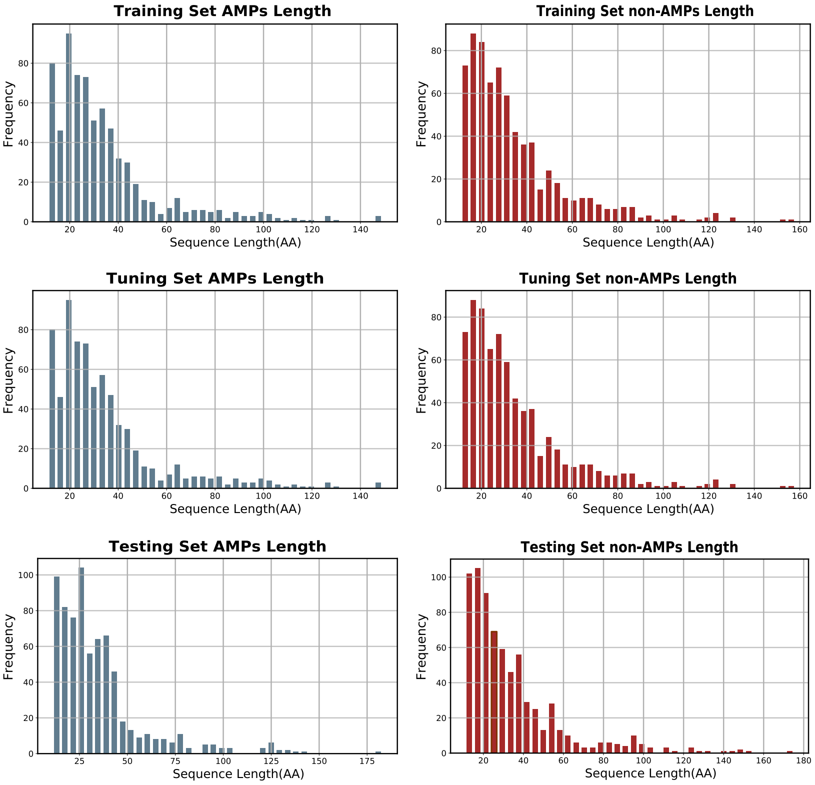

Figure S1: Sequence length distributions of AMPs and non-AMPs

2 Experimental setup and runtime performance

The experiments are conducted on an Intel core i7 laptop with an eight core 2.2GHz processor and 8GB of RAM. The deep neural network is built on Keras vr.2.1.5 using a GPU-based TensorFlow vr.1.6.0 backend. Training takes approximately 10 minutes with the training set, and 15 minutes using all of the data and 3h for 10-fold CV. It takes < 1 minute to run a trained neural network on the testing set.

3 The connections and shapes of each layer

Figure S2 shows the shapes and connections of each layer in ACEP model. The yellow module, the blue module and the red module correspond to feature generating regions R1, R2 and R3, respectively. The green module corresponds to the feature fusion region R4; the purple module corresponds to the sigmoid node that outputs the prediction results.

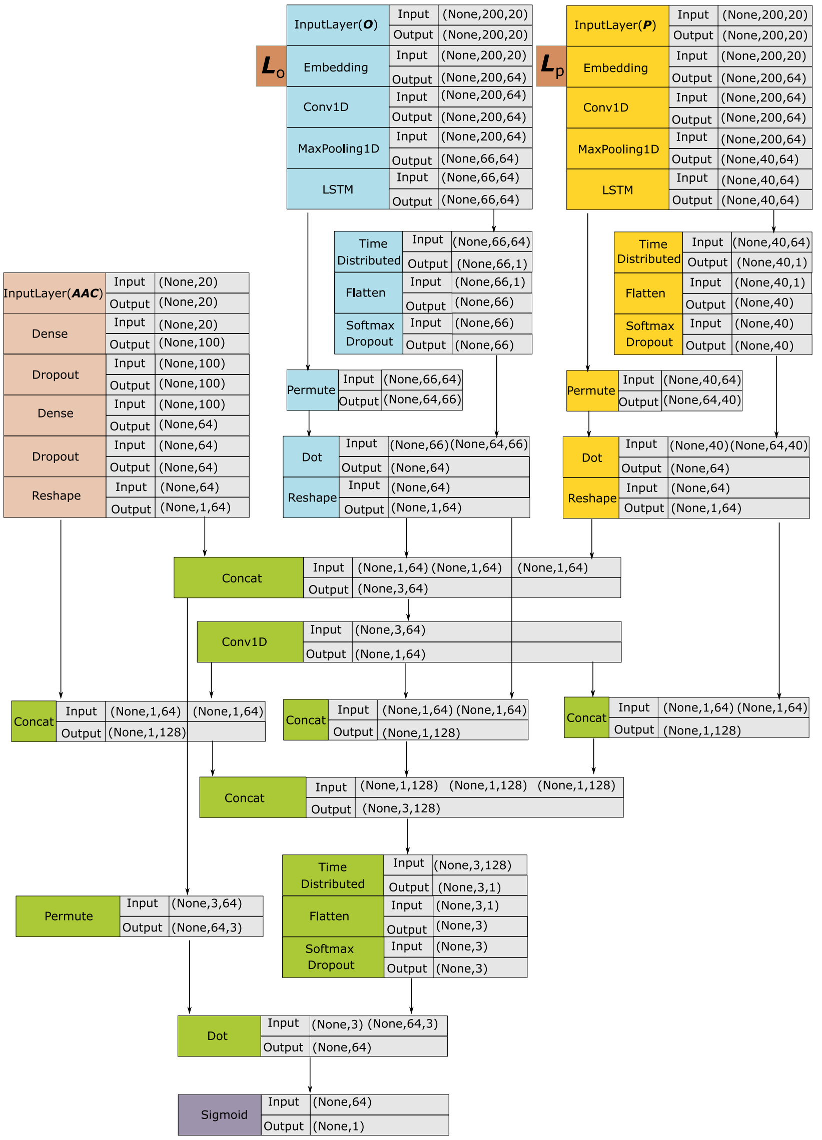

Figure S2: The shapes and connections of each layer in ACEP model.

4 Misclassified AMPs

Table S1: False negative AMP sequences.

APD Identifier	Sequence
AP02360	MVALLKSLERRRLMITISTMLQFGLFLIALIGLVIKLIELSNKK
AP01802	RPWAGNGSVHRYTVLSPRLKTQ
AP01343	TESYFVFSVGM
AP02702	LRHKVYGYCVLGP
AP01969	GPVGLLSSPGSLPPVGGAP
AP02351	QKIAEKFSGTRRG
AP01339	FLSFPTTKTYFPHFDLSHGSAQVKGHGAK
AP02805	VVYTLKRNGRTLYGF
AP02666	AVAGEKLWLLPHLLKMLLTPTP
AP02517	PPPVIKFNRPFLMWIVERDTRSILFMGKIVNPKAP
AP01975	KQIMTQFFNFARSPAVKD
AP02269	CVHWMTNTARTACIAP
AP02624	EVASFDKSKLK
AP02367	INLKAIAALARNY
AP02743	MGYGDIMKVDTSGASMKTAGQDRLTYAGVAASNTMAQTDLGRMNNYKAIIQRVGGKKDVDPAII AGIISRESRAGNVLVNGWGDNGNAWGLMQVDKRYHTPQGGWNSEEHLSQGTDIISFIKQVQGKF PSWTAEQQLKGGIAAYNIGLGGVQTYERMDVGTTGDDYSSDVVARAQWYKSQGGF
AP00140	SQLGDLGSGAGQGGGGGGSIRAAGGAFGKLEAAREEEFFYKKQKEQLERLKNDQIHQAEFHHQQI KEHEEAIQRHKDFLNNLHK
AP00520	DSHAKRHHGYKRKFHEKHHSHRGYRSNYLYDN
AP00480	VGIGTPIFSYGGGAGHVPEYF
AP01230	DGNDGQAELIAIGSLAGTFISPGFGSIAGAYIGDKVHSWATTATVSPSMSPSGIGLSSQFGSGRGTSSA SSSAGSGS
AP01233	QKKPPRPPQWAVGHFM
AP00806	HHQELCTKGDDALVTELECIRLRISPETNAAFDNAVQQLNCLNRACAYRKMCATNNLEQAMSVYF TNEQIKEIHDAATACDPEAHHEHDH
AP01831	ILPFVAGVAAMEMEHVYCAASKKC
AP01195	KRGSGWIATITDDCPNSVFVCC
AP01724	GTPGFQTPDARVISRFGFN
AP01205	STPVLASVAVSMELLPTASVLYSDVAGCFKYSAKHHC
AP00812	FAEPLPSEEEGESYSKEPPEMEKRYGGFM
AP01941	CVHWQTNTARTSCIGP
AP02895	SMATPHVAGAAALILSKHPTWTNAQVRDRLESTATYLGNSFYYGK
AP02250	MKTILRFVAGYDIASHKKKTGGYPWERGKA
AP01004	DWTAWSALVAAACSVELL
AP01326	SKGKKANKDVELARG
AP02783	ISQSDAILSAIWSGIKSLF
AP00560	TTLTLHNLCPYPVWWLVTPNNGGFPIIDNTPVVLG
AP01794	FVDLKKIANIINSIF
AP02197	PAAAAQAVAGLAPVAAEQ
AP00749	EADEPLWLYKGDNIERAPTTADHPILPSIIDDVKLDPNRRYA
AP02321	TNYGNGVGVPDAIMAGIIKLIFIFNIRQGYNFGKKAT
AP00666	EGGGPQWAVGHFM
AP00175	DSHEERHHGRHGHHKYGRKFHEKHHSHRGYRSNYLYDN
AP02028	KRKCPKTPFDNTPGAWFAHLILGC
AP02249	FISQIISTAHI
AP00027	ITPATPFTPAIITEITAAVIA
AP01624	HAEHKVKIGVEQKYGQFPQGTEVTYTCSGNYFLM
AP00998	ALPKKLKYLNLFNDGFNYMGVV
AP01379	ILENLLARSTNEDREGSIFDTGPIRRPKPRPRPRPEG
AP02858	GATPEDLNQKLS
AP00990	RNCESLSHRFKGPCTRDSN
AP01632	ATPATPTVAQFVIQGSTICLVC
AP00754	ETESTPDYLKNIQQQLEEYTKNFNTQVQNAFDSDKIKSEVNNFIESLGKILNTEKKEAPK
AP00741	PITYLDAILAAVRLLNQRISGPCILRLREAQPRPGWVGTLQRRREVSFLVEDGPCPPGVDCRSCEPGA LQHCVGTVSIEQQPTAELRCRPLRPQ
AP02193	YSKSLPLSVLNP
AP02030	MQIFVKTLTGKTITLEVEPSDTIENVKAKIQDKEGIPPDQQRLIFAGKQLEDGRTLSDYNIQKESTLHL VLRLR
AP00996	ISLEICAIFHDN
AP02072	MSNTQAERSIIGMIDMFHKYTRRDDKIDKPSLLTMMKENFPNFLSACDKKGTNYLADVFEKKDKN EDKKIDFSEFLSLLGDIATDYHKQSHGAAPCSGGSQ

5 Additional Data

In Additional_Data.xlsx, sheet 1 is the amino acid embedding tensor with evolutionary information; sheet 2 is the attention score in the CVCA layer; sheet 3 is the attention score in the LSTM layer; and sheet 4 is the motifs discovered by ACEP.

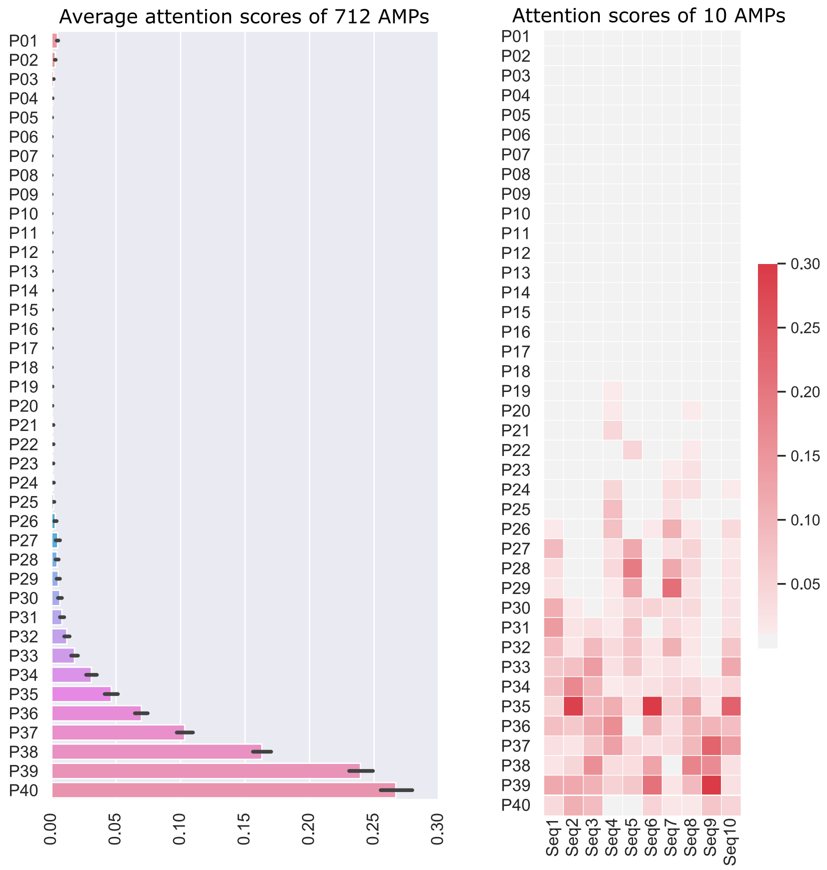

Figure S3: The attention scores of different parts of sequences

References

Altschul, S. F. *et al.* (1997). Gapped BLAST and PSI-BLAST: a new generation of protein database search programs. *Nucleic Acids Res.*, **25**(17), 3389–3402.

Hochreiter, S. and Schmidhuber, J. *et al.* (1997). Long short-term memory. *Neural Comput.*, **9**(8), 1735–1780.

LeCun, Y. *et al.* (2015). Deep learning. *Nature*, **521**(7553), 436–444.

Lloyd, S. *et al.* (1982). Least squares quantization in PCM. *IEEE Trans. Inf. Theory*, **28**(2), 129–137.

Van der Maaten, L. and Hinton, G. *et al.* (2008). Visualizing data using t-sne. *J. Mach. Learn. Res.*, **9**(Nov), 2579–2605.

Qiang, X. *et al.* (2018). CPPred-FL: a sequence-based predictor for large-scale identification of cell-penetrating peptides by feature representation learning. *Briefings Bioinf.*

Rousseeuw, P. J. *et al.* (1987). Silhouettes: a graphical aid to the interpretation and validation of cluster analysis. *J. Comput. Appl. Math.*, **20**, 53–65.

Veltri, D. *et al.* (2018). Deep learning improves antimicrobial peptide recognition. *Bioinformatics*, **34**(16), 2740–2747.

Wang, G. *et al.* (2015). APD3: the antimicrobial peptide database as a tool for research and education. *Nucleic Acids Res.*, **44**(D1), D1087–D1093.

Wang, J. *et al.* (2017). POSSUM: a bioinformatics toolkit for generating numerical sequence feature descriptors based on pssm profiles. *Bioinformatics*, **33**(17), 2756–2758.
